# Diabetes and MRSA Infections: A comparative analysis in an Indian tertiary healthcare setting

**DOI:** 10.1016/j.infpip.2024.100372

**Published:** 2024-05-22

**Authors:** M. Sagiraju, R. Prasad, A. Gopi

**Affiliations:** a Kempegowda Institute of Medical Sciences, Bangalore, India; b Centre for Brain Research, Indian Institute of Science, Bangalore, India

**Keywords:** MRSA, Diabetes, Antibiotic resistance, Healthcare setting and infection control

Dear Editor,

Methicillin-resistant *Staphylococcus aureus* (MRSA) presents a significant global public health challenge, linked to high morbidity and mortality. Initially identified as a prevalent hospital-acquired pathogen, MRSA is now a concern in healthcare and community settings, particularly in developing countries [1]. Studies indicate that MRSA isolation rates in India from outpatients, ward inpatients, and ICU are 27%, 49%, and 47%, respectively [1].

Nosocomial infections caused by MRSA significantly burden healthcare systems, leading to worse patient outcomes, extended hospital stays, increased costs, and higher mortality [2]. MRSA spread has prompted extensive research in various settings, focusing on different demographic groups. However, the prevalence in Indian diabetic patients, a group potentially at higher MRSA colonisation risk [3], remains understudied.

MRSA screening is crucial for controlling nosocomial infections, but in developing countries, resource constraints make universal surveillance challenging. Targeted surveillance, particularly for high-risk groups like diabetic patients, could be a viable alternative.

Our study aimed to determine the prevalence of *S. aureus* and MRSA in diabetic patients compared to non-diabetics with bacterial infections in a major healthcare centre in South India. The study included randomly selected hospitalised patients with bacterial infection out of which we selected 160 diabetic patients and 160 non-diabetics. Exclusion criteria included hospitalisation in the past year, recent antibiotic use (oral within 3 days, intramuscular within 28 days), and viral or fungal infections.

Samples were taken from diabetic and non-diabetic patients (Table I) and were cultured using the BacT/Alert system (manufactured by bioMérieux India Pvt. Ltd in Delhi, India, Software: BacT/Alert 3D Select Software) for five days. If growth was found on the culture plates, it was subcultured on 5% sheep blood agar and MacConkey agar. The cultures on sheep blood agar were done at 37 degrees Celsius with 5–10% carbon dioxide concentration for 48–72 hours. Identification and Antibiotic susceptibility testing for all plates that showed growth was done according to the Clinical and Laboratory Standard Institute guidelines with the help of an automated identification system called VITEK 2 – Compact Systems (manufactured by bioMérieux India Pvt. Ltd in Delhi, India Software: MYLA). It has an accuracy of 98.3% for identifying species and 98% for detecting antimicrobial resistance [4]. Testing was performed using a GP card for ID and a GP 628 card for AST. S. aureus strains ATCC 25923 (MSSA), ATCC 29213 (MSSA), and ATCC 43300 (MRSA) were used as reference strains in oxacillin susceptibility testing as recommended by the Clinical and Laboratory Standards Institute. Resistance was tested for Benzylpenicillin, Cefoxitin, Ciprofloxacin, Clindamycin, Daptomycin, Erythromycin, Gentamicin, Gentamicin High Level, Inducible Clindamycin Resistance, Levofloxacin, Linezolid, Nitrofurantoin, Oxacillin, Rifampicin, Teicoplanin, Tetracycline, Tigecycline, Trimethoprim/Sulfamethoxazole and Vancomycin. The AST results for S. aureus were analysed to determine the prevalence of oxacillin and vancomycin resistance, identifying MRSA and VRSA, respectively, while also assessing resistance patterns to other antibiotics (Figure I).

**Table I:** Characteristics of participants classified by diabetic status

Characteristic	Overall, *N* = 320^a^	Non - diabetic, *N* = 160^a^	Diabetic, *N* = 160^a^	*P*-value^b^
**Age**	52 (16)	46 (16)	58 (12)	<0.001
**Sex**				0.3
Male	213 (67%)	102 (64%)	111 (69%)	
Female	107 (33%)	58 (36%)	49 (31%)	
**Sample**				>0.9
Blood	15 (4.7%)	7 (4.4%)	8 (5.0%)	
CSF	2 (0.6%)	1 (0.6%)	1 (0.6%)	
Fluid	8 (2.5%)	4 (2.5%)	4 (2.5%)	
Pus	203 (63%)	102 (64%)	101 (63%)	
Sputum	30 (9.4%)	15 (9.4%)	15 (9.4%)	
Urine	62 (19%)	31 (19%)	31 (19%)	
***Staphylococcus aureus***				0.12
Absent	240 (75%)	126 (79%)	114 (71%)	
Present	80 (25%)	34 (21%)	46 (29%)	
**MRSA**				0.03
Absent	278 (87%)	146 (91%)	132 (83%)	
Present	42 (13%)	14 (8.8%)	28 (18%)	
**VRSA**				0.2
Absent	301 (94%)	153 (96%)	148 (93%)	
Present	19 (5.9%)	7 (4.4%)	12 (7.5%)	

Note. MRSA Methicillin-resistant Staphylococcus Aureus, VRSA Vancomycin-Resistant Staphylococcus Aureus.

a Mean (SD); n (%).

b Welch Two Sample t-test; Pearson's Chi-squared test; Fisher's exact test.

**Figure I:**
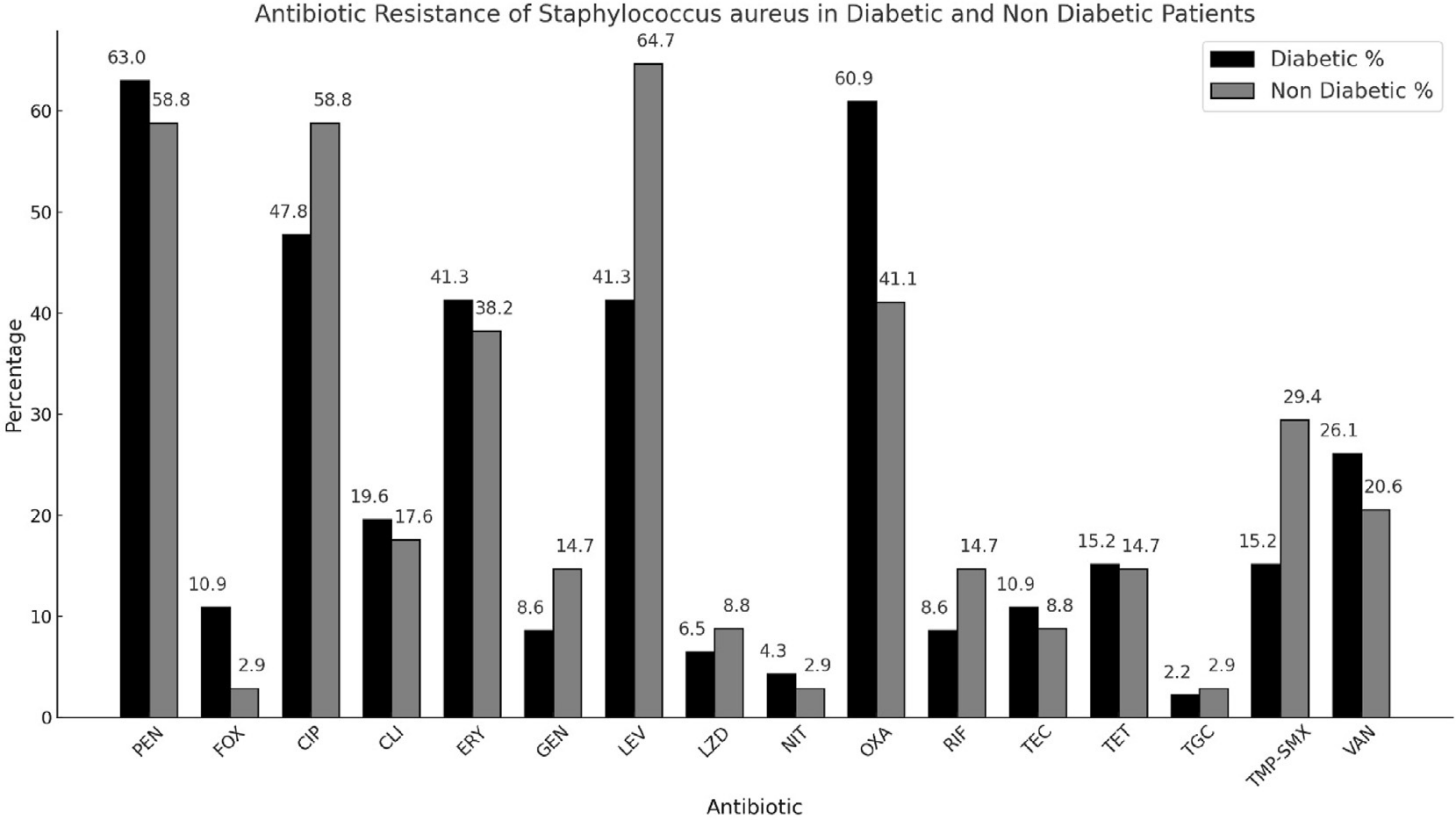
Antibiotic resistance of Staphylococcus aureus in diabetic and non diabetic patients. Abbreviation: PEN Benzylpenicillin, FOX Cefoxitin, CIP Ciprofloxacin, CLI Clindamycin, ERY Erythromycin, GEN Gentamycin, LEV Levofloxacin, LZD Linezolid, NIT Nitrofurantoin, OXA Oxacillin, RIF Rifampicin, TEC Teicoplanin, TET Tetracycline, TGC Tigecycline, TMP-SMX Trimethoprim/Sulfamethoxazole, VAN Vancomycin.

All the statistical analyses were performed on R (version 4.3.2), *P*-values of < 0.05 were considered statistically significant.

Diabetic patients had a significantly increased prevalence of MRSA (17.5% vs. 8.8%, *P*<0.05) even though there was no difference in the prevalence of *Staphylococcus aureus* (Table I). In addition, demographic characteristics of the patients by diabetic status revealed significant differences in age with diabetic patients being older than non-diabetics (59 vs. 44.5 years, *P*<0.001). No significant difference was found in the prevalence of Vancomycin-Resistant *S. aureus* (VRSA) between diabetic patients and non-diabetics.

We looked at the association of diabetes with MRSA utilizing multivariable binary logistic regression adjusting for age and sex which revealed an increased odds ratio of 2.24 (95% CI: 1.09–4.82, *P*-value=0.032). This is potentially due to both innate immune response defects and dysfunctions of the adaptive immune response that are thought to be responsible for the immune system weakness against invading pathogens in diabetic patients [5]. Chronic wounds often occur in patients with diabetes mellitus due to the impairment of wound healing and these often need to be treated with antibiotics. Patients with type 2 diabetes mellitus are hospitalised at a considerably high rate for causes directly related to diabetes complications and stay longer in hospital [6] which increases their risk of acquiring MRSA as it is mainly transmitted in hospital environments.

Our study's prevalence rate of MRSA (13%) is higher than other studies done on diabetic populations such as those in China (4.16%) [3], and Turkey (9.9%) [7]. This could be because our study was exclusively done in the hospital setting. Concerningly, 25% of MRSA isolates were resistant to vancomycin, possibly due to its overuse in MRSA treatment. This could lead to highly resistant 'superbugs', highlighting the need for prudent antibiotic policies and infection control in hospitals.

Our findings raise questions about the vulnerability of diabetic patients to MRSA, the adequacy of current hospital protocols, and the need for effective barriers against MRSA spread in Indian hospitals. The higher MRSA risk in diabetic patients suggests a need to revise screening and treatment protocols, including routine MRSA screening for diabetic patients, enhanced transmission precautions, and possibly isolated wards for diabetic patients. Efforts towards early MRSA identification, transmission limitation, and reducing hospital admissions can significantly reduce societal disease burden.

## Conflict of interest

All authors declare no conflict of interest. All co-authors consent to the manuscript content and there is no financial interest to report. We certify that the submission is original work and is not under review at any other publication.

## Funding

The research received a grant from the 10.13039/501100001411Indian Council of Medical Research (10.13039/501100001411ICMR) as part of the 10.13039/501100001411ICMR 10.13039/100007120STS program. 10.13039/501100001411ICMR Research Grant No. 2020-04461. ICMR had no role in the study design, data collection and analysis, decision to publish, or preparation of the manuscript.

## Ethics statement

The study was approved by The Institutional Ethics Committee of KEMPEGOWDA INSTITUTE OF MEDICAL SCIENCES (Reference number: KIMS/IE/A001-2020).
